# Imidazolopiperazine (IPZ)-Induced Differential Transcriptomic Responses on *Plasmodium falciparum* Wild-Type and IPZ-Resistant Mutant Parasites

**DOI:** 10.3390/genes14122124

**Published:** 2023-11-24

**Authors:** Laurent Dembele, Antoine Dara, Mohamed Maiga, Fatoumata O. Maiga, Djeneba Cissoko, Abdoulaye A. Djimde

**Affiliations:** 1 Malaria Research and Training Center, Faculty of Pharmacy, Université des Sciences, des Techniques et des Technologies de Bamako (USTTB), DEAP Point G, Bamako P.O. Box 1805, Mali; tonydara@icermali.org (A.D.); mohamedmaiga@icermali.org (M.M.); fatouomaiga@icermali.org (F.O.M.); djenebacissoko@icermali.org (D.C.); adjimde@icermali.org (A.A.D.); 2 African Center of Excellence in Bioinformatics (ACE), Bamako P.O. Box 1805, Mali; 3 Novartis Institute for Tropical Diseases, 10 Biopolis Road, #05-01 Chromos, Singapore 138670, Singapore

**Keywords:** malaria, imidazolopiperazine, mechanism, resistance, treatment

## Abstract

Imidazolopiperazine (IPZ), KAF156, a close analogue of GNF179, is a promising antimalarial candidate. IPZ is effective against *Plasmodium falciparum* and *Plasmodium vivax* clinical malaria in human with transmission blocking property in animal models and effective against liver stage parasites. Despite these excellent drug efficacy properties, in vitro parasites have shown resistance to IPZ. However, the mechanism of action and resistance of IPZ remained not fully understood. Here, we used transcriptomic analysis to elucidate mode of action of IPZs. We report, in wild-type parasites GNF179 treatment down regulated lipase enzymes, two metabolic pathways: the hydrolysis of Phosphoinositol 4,5-bipohosphate (PIP2) that produce diacyglycerol (DAG) and the cytosolic calcium Ca^2+^ homeostasis which are known to be essential for *P. falciparum* survival and proliferation, as well for membrane permeability and protein trafficking. Furthermore, in wild-type parasites, GNF179 repressed expression of Acyl CoA Synthetase, export lipase 1 and esterase enzymes. Thus, in wild-type parasites only, GNF179 treatment affected enzymes leading lipid metabolism, transport, and synthesis. Lastly, our data revealed that IPZs did not perturb known IPZ resistance genes markers *pfcarl*, *pfact,* and *pfugt* regulations, which are all instead possibly involved in the drug resistance that disturb membrane transport targeted by IPZ.

## 1. Introduction

Following *P. falciparum* resistance to chloroquine and sulphadoxine–pyrimethamine, artemisinin-based combination therapies (ACTs) are currently used as front-line treatment for uncomplicated malaria [1]. Despite the remarkable impact of ACTs in reducing malaria cases and deaths [2], drug resistance is compromising their use [3]. The emergence of the resistance to ACTs has continued to evolve and spread worldwide [4,5,6]. Thus, the threat of widespread resistance to *ACTs* renewed the urgency for identifying novel effective antimalarial drugs against Plasmodium falciparum malaria. A novel class of antimalarial drug candidate, the imidazolopiperazine (IPZ) compound KAF156, appeared as a promising alternative treatment of human malaria to combat rapid emergence of ACTs [7].

IPZ was identified through a cell-based phenotypic screen [8]. IPZs (KAF156 and its close analogue GNF179) have shown excellent inhibitory activity against a broad range of stages of the *Plasmodium* life cycle, including liver stage schizonts, across asexual blood stages as well as gametocytes [8,9]. Our previous report showed that GNF179 potently inhibited artemisinin resistant parasites bearing *PfKelch13* (K13) propeller mutations and displayed fastest cidal activity against the blood stage schizont parasites [10]. IPZ KAF156 is currently in phase IIb clinical trials, where it has been efficacious in patients with *P. falciparum* and *P. vivax* malaria [7]. As a great promising antimalarial candidate, IPZ was also efficacious in patients infected with *P. falciparum* bearing K13 propeller mutations [7]. In animal models and mosquito feeding assays, IPZs have shown good prophylactic and gametocytocidal activity [11]. KAF156 is well tolerated in human with a half-life elimination of 48.7 ± 7.9 h [7]. If approved as a treatment for malaria, IZPs have a potential to represent next-generation antimalarial drugs with additional excellent properties such as malaria prevention and transmission blocking [11]. 

Previous reports have shown the *P. falciparum* cyclic amine resistance locus gene (*pfcarl*), the *P. falciparum* Acetyl CoA Transporter (*pfact*), and the *P. falciparum* UGT_galactose transporter (*pfugt*) are the markers of IPZs resistance in vitro [12,13]. A recent excellent study has shown that IPZs inhibited protein trafficking affecting lipid hemostasis while mutations in these three transmembrane transporters (*pfcarl*, *pfact,* and *pfugt*) were suggested to be involved in resistance to IPZs [14]. However, the key molecular players and pathways involved in the drug mechanism of action and resistance remained to be identified. Therefore, we performed genome-wide transcriptome analysis on *P. falciparum* Dd2 wild-type and its mutant pfcarl, pfact, and pfugt parasites, which are resistant to IPZs, all treated with GNF179 and compared to their drug-unexposed parasites in order to identify gene expression signatures in response to GNF179 treatment.

## 2. Materials and Methods

Antimalarial drug. Novartis synthesized the compound GNF179 internally [15]. 

Parasites. *P. falciparum* laboratory-adapted parental strain Dd2 (a clone of W2MEF) was used, along with its mutant pfcarl, pfact, and pfugt parasites, which are resistant to IPZs and were generated in house [13]. 

Parasite culture. *P. falciparum* Dd2 and its mutant pfcarl, pfact, and pfugt IPZ-resistant parasites were cultured using standard RPMI 1640-HEPES (Gibco Life Technologies, Singapore) mediums supplemented with 0.5% AlbuMAX and 4% red blood cells (RBCs). RBCs used in this study were obtained from Innovative Research (Novi, MI, USA) (https://www.innov-research.com/collections/human-whole-blood-and-blood-cells (accessed on 27 September 2023)). Parasites were synchronized with 5% d-sorbitol [16] at each cycle of the parasite. For each experiment, parasites were synchronized twice with 5% sorbitol at the ring stage and suspended in the initial volume of growth medium for 38 h incubation under normal growth conditions to reach the young schizont stage.

### 2.1. Drug Treatment for Transcriptomic Sample Preparation

**Figure 1:**
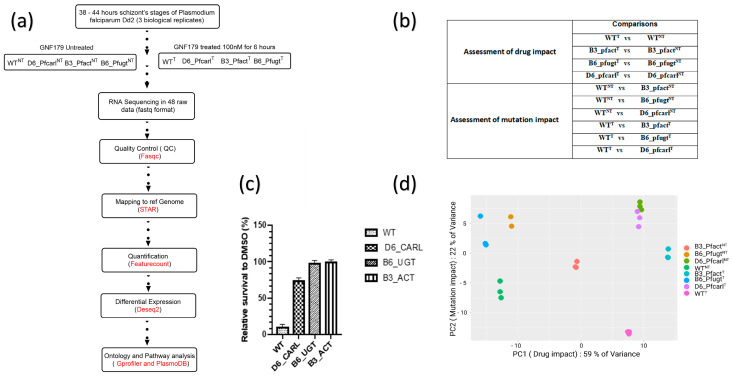
Study design, data analysis and distribution. (**a**) experimental procedure and bioinformatics analysis pipeline; (**b**) samples analysis plan design; (**c**) wild-type (WT) and mutant (B3_Pfact), (B6_Pfugt), (D6_Pfcarl) parasites resistant to IPZ parasites survival under GNF179 (100 nM) in vitro treatment; (**d**) principal component analysis and distribution of normalized expression data of WT and mutant parasites resistant to Imidazolopiperazine in GNF179 (100 nM) treated and untreated conditions showing drug and mutations impacts on gene expressions.

**Figure 2:**
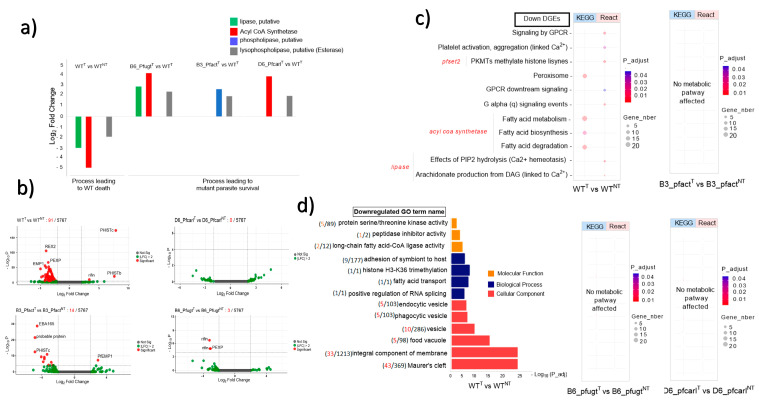
Key genes, metabolic pathways, and biological processes impacted or not by Imidazolopiperazine (IPZ) GNF179 (100 nM) treatment in *P. falciparum* Dd2 wild-type and its mutant parasites resistant to IPZ. (**a**) Genes down regulated in dying wild-type (WT) parasites that were upregulated in surviving mutant (B3_Pfact), (B6_Pfugt), (D6_Pfcarl) parasites resistant to IPZ; (**b**) volcano plot display GNF179 impact on transcriptomic profile between each treated and its untreated homologue parasites.; (**c**) key metabolic pathways affected by GNF179 (100 nM) exposure in WT parasites only not in mutant (B3_Pfact), (B6_Pfugt), (D6_Pfcarl) parasites resistant to IPZ; (**d**) Cellular components, biological process and molecular function disturbed by GNF179 (100 nM) exposure in wild-type GNF179 (100 nM) treated (WT^T^) as compared to not treated (WT^NT^).

Following 38 h incubation, the culture was divided into two schizont samples. One sample was maintained under normal culture conditions with DMSO treatment as a control, while the other sample was exposed to 100 nM GNF179 for 6 h, all in triplicate (Figure 1a). After 6 h of treatment with DMSO and GNF179, the cultures of both samples were washed twice with phosphate buffered saline (PBS) 1× to remove the drug and cellular debris before being stored at −80 °C. Part of the samples were frozen for RNA extraction and the rest were analyzed with HCI (high content imaging) after staining with MitoTracker orange according to our previous report [10] to evaluate the effect of the treatment on parasite survival (Figure 1a and Figure 2b). Three independent experiments with each three technical replicates were used to generate samples for the transcriptomic study (Figure 1a).

### 2.2. mRNA Extraction and Sequencing

The total mRNA was isolated and then converted into cDNA by retro transcription. Illumina universal adapter was used for the preparation of the library. The whole set was sequenced using HiSeq Illumina 3000 1.9 technology to generate 48 reads (Pair-End), either 6 reads per sample of high throughput raw data (NGS) in Fastq format.

### 2.3. Bioinformatics Analysis

RNASeq data analysis pipeline used for the bioinformatics analysis is displayed in (Figure 1a). The 48 fastq files generated on an Illumina platform (HiSeq Illumina 3000 1.9) were first stored on a local server (delgeme.icermali.org). Quality control of the raw data was performed with FastQC v0.11.4. Subsequently, STAR V.2.7.0a was used for indexing and alignment using the *P. falciparum* 3D7 reference genome version ASM276v2. STAR tool takes as input Fastq files [17], the reference genome (ASM276v2) and the annotation file (ASM276v2) that were downloaded from ENSEMBL [18], respectively, as input with multiple options and gives output as *bam* or *sam* files. Quantification of mapped reads was performed by FeatureCounts [19,20]. DESeq2 was used for normalization and differential expression [21,22] via Bioconductor version 3.1.1 package on R version 4.0.5.

The ontology of differentially expressed genes (GO terms) and metabolic pathway analysis were performed in the KEGG and Reactome (React) databases via GProfiler [23,24] and PlasmoDB. The transcriptomic profile of the different parasites was compared to show the mechanism of action and resistance of IPZs. 

### 2.4. Comparison of Normalized Counts

Normalized counts for the three biological replicates of the samples were generated using the median of ratios method, on which Deseq2 (Figures S1–S6) is based. Prism version 9 and t test were then used for statistical analysis of the data.

## 3. Results

### 3.1. P. falciparum Wild-Type and Imidazolopiperazine-Resistant Mutant Parasites Displayed Differential Transcriptomic Response from GNF179 Exposure

Using transcriptomic analyzes of *P. falciparum* Dd2 wild-type and its IPZs-resistant mutant Pfcarl, Pfact, and Pfugt parasites (Figure 1a), we evaluated the effect of GNF179 drug (100 nM) and the mutations impact on these parasite’s genes expression as displayed in (Figure 1b,c). Sequencing reads were of high quality, with an average Phred score above 30 for all base positions (Figure S1a). A principal component (PC) analysis of this data has shown that 58% of gene expression variance was due to drug impact while mutations impact accounted together for 24% of the variance in the gene expressions (Figure 1d). The PC analysis has also shown a good data quality across all biological replicates (Figure S1b). Using these data, we set to assess the overall GNF179 treatment impact on the gene expression in Dd2 wild-type and mutant pfcarl, pfact, and pfugt parasites resistant to IPZs. In this regard, we found that 92 out of the total of 5555 genes were differentially regulated in wild-type parasites GNF179 treated (WT^T^) versus untreated wild-type parasites (WT^NT^) (Figure 2b). Most of the genes, eighty-nine were down regulated (log fold change (LFC) < −2 and *p* value < 0.05) and only three were upregulated (LFC > 2 and *p*-value < 0.05) in the wild-type (WT) parasites (Figure S2). Interestingly, GNF179 treatment did not induce any major alteration in the gene expression across all three mutants (Figure 2b and Figure S2). Thus, mutant parasites resistant to GNF179 were transcriptionally stable in response to GNF179 treatment. 

Having shown that GNF179 had only subtle impact on parasite and only induce transcriptome changes in few targeted genes in both WT and mutant parasites, we set to identify the key molecular players that could be essential for the parasite development and survival against GNF179 treatment. Five criteria were thus defined to identify potential genes of interest: (1) genes must be significantly differentially altered: LFC > 2 or <2 and *p*-value < 0.05; (2) it must be involved in a known essential biological process or metabolic pathway that is key for parasite survival (FDR < 0.05); (3) it should encode a membrane transport or binding protein; (4) it should encode an enzyme; and finally (5) it should be a potential drug target or marker of pathogen drug resistance. Thus, based on above criteria, we identified four genes that were significantly downregulated in wild-type parasites treated with 100 nM of GNF179 that resulted in drastic parasite killing. These were putative lipase (PF3D7_1427100, *p* = 2.43 × 10^−15^), Acyl CoA Synthetase (PF3D7_0401900, *p* = 2.68 × 10^−15^), esterase (PF3D7_1401500, *p =* 5.888457 × 10^−8^), and export lipase 1 enzymes (PF3D7_1001400, *p* = 1.19 × 10^−38^) (Figure 2a). Unlike wild-type parasites, all mutant parasites survived from GNF179 exposure as shown in (Figure 1c). Interestingly, GNF179 induced upregulation of these genes across these three mutant parasites (pfcarl, pfact and pfugt) which are resistant to IPZ (Figure 2a). Strikingly, the identified esterase (*PF3D7_1401500*) has a conserved role for remodeling phospholipids, a major constituent of biological membranes that was downregulated in WT treated with GNF179 while significantly upregulated in all surviving mutant parasites resistant to GNF179 (Figure 1c and Figure 2a). Only in wild-type parasites exposed to GNF179, known key metabolic pathways and biological processes were downregulated (Figure 2c,d). These included fatty acid biosynthesis and degradation by lipid hydrolysis (TAG or PIP2) affecting parasite calcium (Ca^2+^) homeostasis and DAG production (Figure 2c). Gene ontology analysis shows the impact of GNF179 treatment on biological processes associated to fatty acid transport and protein methylation (Figure 2d). Based on the above results, it is tempting to speculate that these four genes play crucial roles in the survival of IPZs mutant parasites against GNF179 treatment with furthermore experimental validation required.

### 3.2. Imidazolopiperazine (IPZ) GNF179 Treatment Did Not Affect Pfcarl, Pfact, and Pfugt Genes Regulation Which Are Associated with Membrane Transport Targeted by IPZ

**Figure 3:**
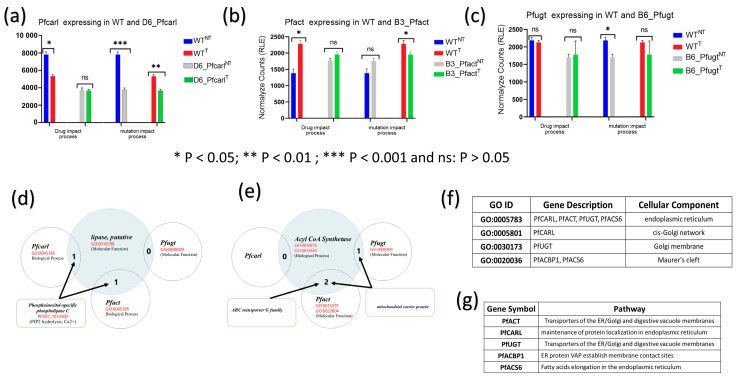
*Pfcarl, Pfact,* and *Pfugt* are not directly targeted by imidazolopiperazine (IPZ) GNF179 but associated with membrane transport. Drug GNF179 (100 nM) and the mutations impact on the genes *Pfcarl, Pfact, Pfugt* conferring resistant to IPZ in the following mutants: (**a**) D6_Pfcarl; (**b**) B3_Pfact and (**c**) B6_Pfugt parasites. (**d**) Common biological process, molecular function between lipase putative and *Pfcarl*, *Pfact* genes. (**e**) Common biological process, molecular function between acyl CoA synthetase (*Pfacs6*) *Pfact* and *Pfugt* genes. (**e**) Common biological process, molecular function between acyl CoA synthetase (*Pfacs6*), acyl-CoA-binding protein 1 (*PfACBP1*) and *Pfact, Pfugt* genes. (**f**) common cellular component between *Pfacs6* and *pfacbp1*, *pfcarl*, and *pfact*. (**g**) acyl CoA synthetase (Pfacs6), acyl-CoA-binding protein 1 (*PfACBP1*), *Pfcarl*, *Pfact*, and *Pfugt* association with membrane transport and involvement in ER-controlled metabolic pathway (Malaria Parasite Metabolic Pathways: MPMP).

Having shown that IPZ GNF179 treatment mainly target various vital and essential cell components, metabolic, and signaling pathways that facilitate protein trafficking, cell exchanges, and proliferation in wild-type parasites only; we set to assess the effect of GNF179 drugs on mutations conferring resistance to IPZ (Figure 3 and Figure S3). In this regard, we performed a comparison of three biological replicates normalized counts across wild-type and mutant parasites using relative log expression (RLE) normalization and a statistic *t*-test (Figure 3a–c).

The expression of *Pfcarl* genes whose mutation S1076I confer resistance to IPZ was significantly downregulated in GNF179 treated wild-type parasites (WT^T^) as compared to the untreated parasites (WT^NT^) (Figure 3a). Interestingly, GNF179 treatment did not affect *Pfcarl* expression in mutant D6_Pfcarl displaying same level of expression in D6_Pfcarl treated (D6_Pfcarl^T^) and untreated (D6_Pfcarl^NT^) (Figure 3a). The serine to isoleucine substitution at position 1076 (S1076I) in Pfcarl itself repressed expression Pfcarl in D6_Pfcarl GNF179 not treated (D6_Pfcarl^NT^) when compared to GNF179 unexposed wild-type (WT^NT^) (Figure 3a). Thus, the mutation S1076I alone resulted in a strong repression of *pfcarl* in D6_Pfcarl^NT^ like in GNF179 treated wild-type (WT^T^) (Figure 3a) with the different following consequences: survival for mutant D6_Pfcarl and death for the GNF179 treated wild-type (WT^T^) (Figure 1c and Figure 3a). Therefore, these results indicated that repression of *pfcarl* expression alone in GNF179 treated wild-type parasites is not responsible for their death (Figure 3a). 

The mutation S1076I itself happened in the untreated (D6_Pfcarl ^NT^) displayed the same impact of repression of *pfcarl* expression as the IPZ drug (Figure 3a). This provides thus evidence that *pfcarl* is not a drug target for IPZ. GNF179 treatment in wild-type (WT^T^) parasites upregulated *pfact* that confer IPZs resistance to B3_Pfact (Figure 3b). However, like *pfcarl* in D6, GNF179 treatment did not affect *pfact* expression in mutant (B3_Pfact^T^) as compared to untreated mutant parasites (B3_Pfact^NT^) (Figure 3b). This stop–gain mutation at position 242 of *pfact* alone did not affect *pfact* regulation in drug unexposed condition (WT^NT^ vs. B3_Pfact^NT^) (Figure 3b). However, stop–gain mutation at position 242 leads to significant down regulation of *pfact* expression in drug-treated conditions (WT^T^ vs. B3_Pfact^T^) (Figure 3b) and thus led to mutant B3_Pfact^T^ survival under GNF179 exposure.

Unlike *pfcarl* and *pfact* that were, respectively, down and upregulated following GNF179 treatment in WT parasites, *pfugt* mutant B6_Pfugt (F37V) was not affected by GNF179 exposure in WT parasites (Figure 3c). The regulation of *pfugt* was not impacted by GNF179 in mutant parasites (B6_Pfugt^T^) treated condition as compared to the untreated (B6_Pfugt^NT^) (Figure 3c). The phenylalanine to valine changes at position 37 (F37V) in *pfugt* gene has down regulated *pfugt* in B6_Pfugt in drug unexposed condition (WT^NT^ versus B6_Pfugt^NT^) which has shown no significant effect in drug treated conditions (WT^T^ versus B6_Pfugt^T^). Thus, the mutation F37V in B6_Pfugt enables the survival of that mutant parasite under treatment condition (B6_Pfugt^T^) (Figure 1c).

Next, we set to assess the molecular players enabling mutant parasite survival from GNF179 treatment. In this regard, and based on the data shown in (Figure 3d,e), we hypothesized that GNF179 could target biological processes in which *pfcarl*, *pfact,* and *pfugt* were involved. The results showed that *pfcarl* and *pfact* or *pfact* and *pfugt* are engaged in common biological processes, respectively, with the putative lipase and Acyl CoA Synthetase (Figure 3d,e). These markers (*pfcarl*, *pfact*, and *pfugt*) conferring drug resistance to IPZs also have similar molecular functions and/ or located in similar cellular compartments with putative lipase and Acyl CoA Synthetase (Figure 3f,g). Out of these three mutations, the stop–gain mutation at position 242 in *pfact* gene (B3_pfact) exhibited the strongest survival (Figure 1c) and found to be associated with both putative lipase and Acyl CoA Synthetase pathways associated to endoplasmic reticulum (ER) function (Figure 3d,e). Furthermore, we observed that commonly affected genes across all mutant parasites were known genes involved in transport and the membrane trafficking of proteins (Figure S4a,b). Similarly, PfACBP1 (PF3D7_1001100.1) was specifically identified as being downregulated in WT^T^ vs. WT^NT^ parasites (Supplementary Figures S1–S6) and in D6_Pfcarl^NT^ vs. WT^NT^ parasites and appears to be localized with PfACS6 in Maurer cells and plays a function associated with protein trafficking in infected red blood cells (Figure 3f,g).

Taken together, these results suggest that Acyl CoA synthetase, PfACBP1, pfcarl, pfact, and pfugt are located in the same cell compartment or involved in the same biological process that appears to be disrupted by IPZs (Figure 3).

### 3.3. Key Uncommon Metabolic Pathways and Biological Process Disturbed by GNF179 between Wild and IPZ-Resistant Parasites in Treated and Untreated Conditions

In untreated conditions, when compared to resistant parasites, wild-type parasites essentially showed that the fatty acid transport process downregulated only in wild-type (WT^NT^) vs. B3_PfACT^NT^ and wild-type (WT^NT^) vs. D6_PfCARL^NT^ (Figure S5a,c), while in wild-type (WT^NT^) vs. B6_PfUGT^NT^, no similar process was impacted (Figure S5b). In treated conditions, the same process is upregulated in the wild-type (WT^T^) vs. B6_PfUGT^T^ and wild-type (WT^T^) vs. D6_PfCARL^T^ comparisons (Figure S5e,f), except in the wild-type (WT^T^) vs. B3_PfACT^T^ comparison where processes associated with gametogenesis were impacted (Figure S5d). In addition to the fatty acid transport processes, we observed the upregulation of genes coding protein phosphorylation and translation elongation only in wild-type (WT^T^) vs. B6_PfUGT^T^ (Figure S5e) that are all essential for protein synthesis and maturation. Individually, each resistant parasite had its own specificity, which was either related to protein phosphorylation, gametogenesis, or cytoadherence (Figure S5).

Key metabolic pathways were affected only in two comparisons (Figure S6), with lipid metabolism downregulated in wild-type (WT^NT^) vs. B3_PfACT^NT^ (Figure S6c) and upregulated in wild-type (WT^T^) vs. B6_PfUGT^T^ (Figure S6e). No metabolic pathway was affected in the other comparisons (Figure S6a,b,d,f). Taken together, these results suggest that resistance to IPZs is globally linked to lipid metabolism, in particular fatty acid transport.

## 4. Discussion

To survive and proliferate, *Plasmodium* has created a parasitophorous vacuole, a translocon complex called PTEX, and Maurer’s cleft in the host cell [25,26]. Thus, the parasite renovates the permeability, stiffness, metabolism process, and membrane characteristics of the infected cells to meet its needs. This complex network of tightly regulated trafficking is crucial for the parasite survival and proliferation in the host cells and is therefore a high-profile and an excellent target for antimalarial drugs [27,28,29]. 

Our data have shown that when wild-type parasites were treated with GNF179, four enzymes and vital metabolic pathways genes were significantly downregulated (Figure 2a). These enzymes included two lipid metabolism enzymes: a lipase and an Acyl CoA Synthetase and the third enzyme was an esterase remodeling phospholipid (Figure 2a). However, none of these vital metabolic pathways and key enzymes were impacted by GN179 treatment across the different mutant pfcarl, pfact, and pfugt parasites resistant to GNF179 (Figure 2a–c). Lipids play a major role in the regulation of protein trafficking and membrane dynamics [30,31,32]. Lipases are enzymes that hydrolyze lipids yielding hydrolysis products that vary depending on the nature of the lipid [33,34]. The repression of this lipase in our data (Figure 2a) seems to result in a defect in Diacylglycerol (DAG) production due to the failure of the hydrolysis of Triacylglycerol (TAG) and or PIP2 (Figure 2d). Indeed, in apicocomplexa, PIP2 is hydrolyzed by phospholipase C into Phosphoinositol 1,4,5 Biphosphate (IP3), and DAG [35]. IP3 must bind to ER receptors (IP3R) to release calcium from the ER in eukaryotes; however, these types of receptors have not been identified in *P. falciparum* to date [35]. Despite the lack of identification of its receptor, the increase of IP3 in the cytosol is known in *P. falciparum* to promote Ca^2+^ release from the ER [36]. A defect in PIP2 hydrolysis could therefore disrupt the activity of the Ca^2+^ dependent protein kinases (CDPKs) and protein kinase C (PKC) (Figure 2c,d), which are known to be involved in protein phosphorylation. 

DAG, a phospholipid precursor in *P. falciparum*, is generally produced by the hydrolysis of TAG or PIP2 by lipase or acylation of monoacylglycerol (MAG) by Acyl CoA [37]. Interestingly, GNF179 treatment significantly induced the downregulation of Acyl CoA Synthetase expression in wild-type parasites while upregulated in all mutant parasites resistant to GNF179 (Figure 2a). The strong repression of Acyl CoA Synthetase in wild-type could be associated with the death of those parasites treated with GNF179 (Figure 2a,b). Acyl CoA Synthetase is a ligase that is also involved in fatty acids’ biosynthesis and activation. The activated fatty acids are involved in protein transport, enzyme activation, cell signaling, and transcription control [38]. Acyl CoA Synthetase enzyme also serves as a substrate for β-oxidation in peroxisomes and mitochondria in eukaryotes [39]. Our results also suggest that GNF179 did not directly target *pfcarl*, *pfact,* and *pfugt* genes that all indeed involved a resistance process disrupting the membrane trafficking (Figure 3).

A recent study based on genomic, biochemical and metabolic analysis suggests that GNF179 targets the intracellular secretory pathway and causes ER stress by preventing the sorting, folding, and blocking protein trafficking [14]. The same study [14] also reported that genes involved in lipid metabolism may induce resistance to IPZs. Interestingly, in addition to that study, our data provided important details on the key molecular players that might be involved in sorting, folding, and blocking protein trafficking. These key molecular players would include Acyl CoA Synthetase, esterase remodeling lipids and lipase enzymes whose upregulation may lead to mutant parasites survival following GNF179 treatment while the repression of these genes in wild-type parasites suggested to contributed to their death (Figure 2a). By exploring all the genes differentially expressed in the impact mutation comparisons, we found that the fatty acid transport process or the lipid metabolism pathway were regulated in almost all resistant parasites (Figures S5 and S6.) Thus, on the basis of these results, we hypothesize that resistance to IPZs is associated with a lipid metabolism-related process, probably fatty acid transport, and that this process appears to impact membrane protein trafficking. Together, these findings will help in advancement of mechanistic understanding of GNF179 effects and resistance in *P. falciparum.*

## 5. Conclusions

This gene expression study represents a major step forward in identifying the mechanism of action and resistance of IPZs. It provides genes and key metabolic pathways to guide experimental studies toward validating the hypotheses surrounding the mechanism of action and resistance of IPZs.

## Data Availability

All data generated are included in this published article and its supplementary information file. All the Linux and R codes that were used for all the bioinformatics analysis are available on GitHub https://github.com/Diango700 accessed on 29 November 2023.

## Supplementary Materials

The following supporting information can be downloaded at: https://www.mdpi.com/article/10.3390/genes14122124/s1, Figure S1: Quality Control and Pre-processing of the raw reads; Figure S2: Transcriptomic profile of the drug impact in wild type and mutant (B3_Pfact), (B6_Pfugt), (D6_Pfcarl) parasites resistant to IPZ in GNF179 (100 nM) treated as compared to untreated conditions; Figure S3: Transcriptomic profile of the mutation impacts in (B3_Pfact), (B6_Pfugt), (D6_Pfcarl) parasites resistant to IPZ compared to wild type; Figure S4: Export protein affected on common mutant parasites in treated and untreated con-ditions; Figure S5: Cellular components, biological process and molecular function disturbed by GNF179 between Wild type and IPZ resistant parasite in GNF179 (100 nM) treated and un-treated condition; Figure S6: Key metabolic pathways affected by GNF179 between Wild type and IPZ resistant parasite in GNF179 (100 nM) treated and untreated condition.
