# Supplementary material for: Imidazolopiperazine (IPZ)-Induced Differential Transcriptomic Responses on Plasmodium falciparum Wild-Type and IPZ-Resistant Mutant Parasites
Source: Genes (Basel). 2023 Nov 24;14(12):2124. doi: 10.3390/genes14122124 (PMC10743112; doi:10.3390/genes14122124)
Supplement: Supplementary file 1 [file genes-14-02124-s001.zip › genes-2664063-supplementary.pdf]

Supplementary Figures

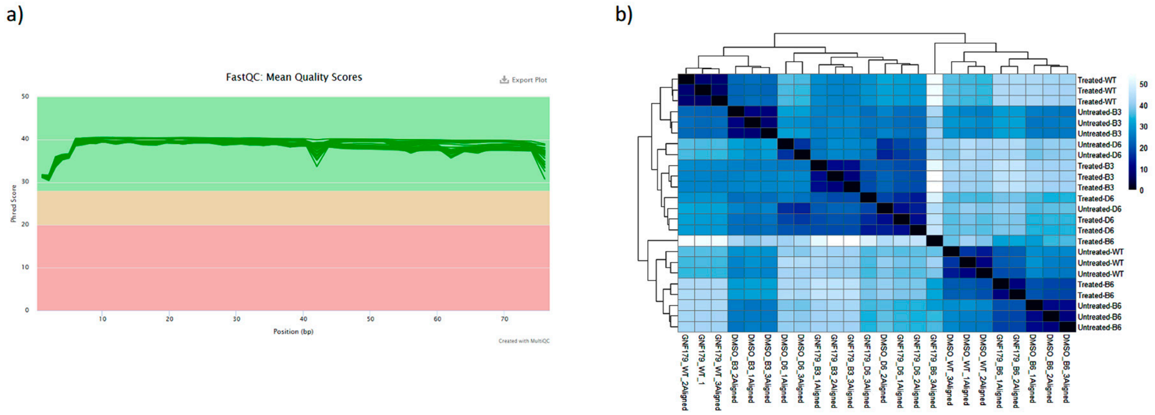

Figure S1. Quality Control and Pre-processing of the raw reads. Quality control of reads by the Fastqc tool which details the quality score for 48 reads. Green = goodquality of the reads, dark Yellow = medium quality of the reads and red = bad quality of the reads. **b)** heatmap plot to indicate similarity between biologic replicate based on Euclidian Distance.

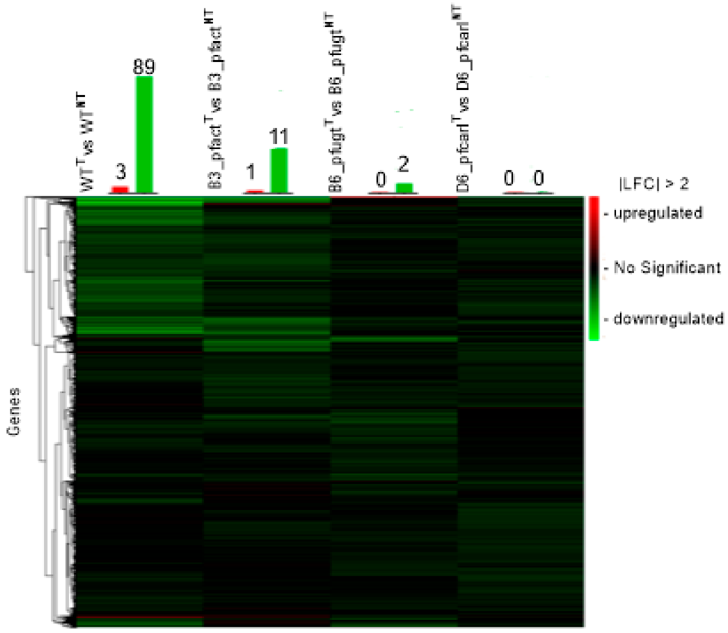

Figure S2. Transcriptomic profile of the drug impact in wild type and mutant (B3_Pfact), (B6_Pfugt), (D6_Pfcarl) parasites resistant to IPZ in GNF179 (100 nM) treated as compared to untreated conditions. Heatmap plot showing upregulate (red) and downregulate (green) genes in each parasite in GNF179 (100 nM) treated as compared to not treated.

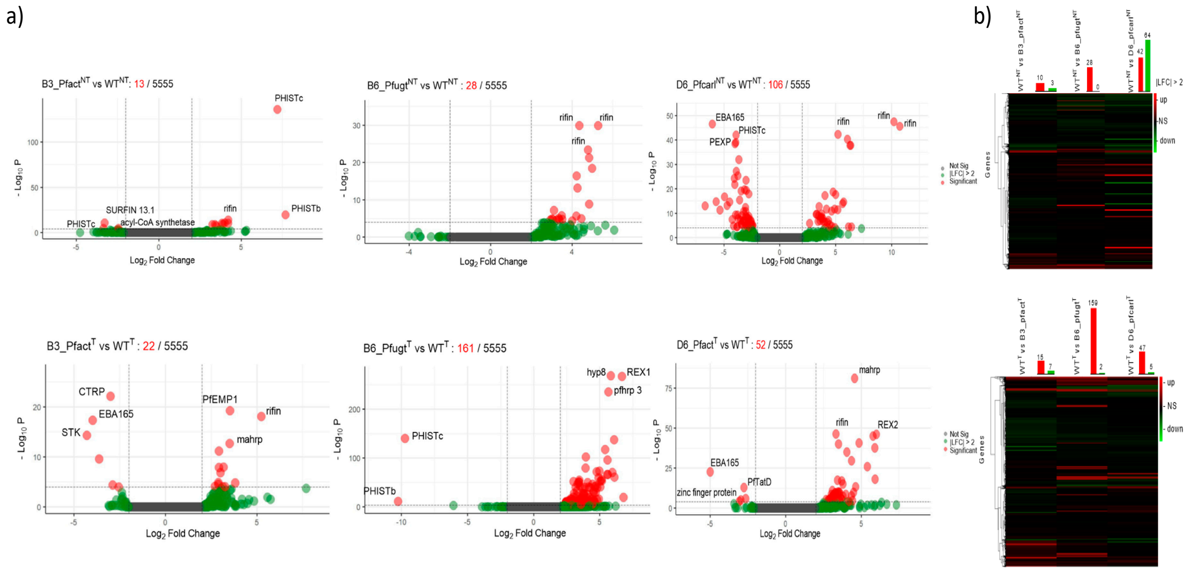

Figure S3. Transcriptomic profile of the mutation impacts in (B3_Pfact), (B6_Pfugt), (D6_Pfcarl) parasites resistant to IPZ compared to wild type. a) volcano plot displaying mutation impact on the transcriptomic profile between each mutant parasite compared to WT in treated and untreated conditions. **b)** heatmap plot showing number of upregulate (red) and downregulate (green) genes in each mutant (B6_Pfugt), (D6_Pfcarl) parasites resistant to IPZ compared to wild type in GNF179 (100 nM) treated and not treated conditions

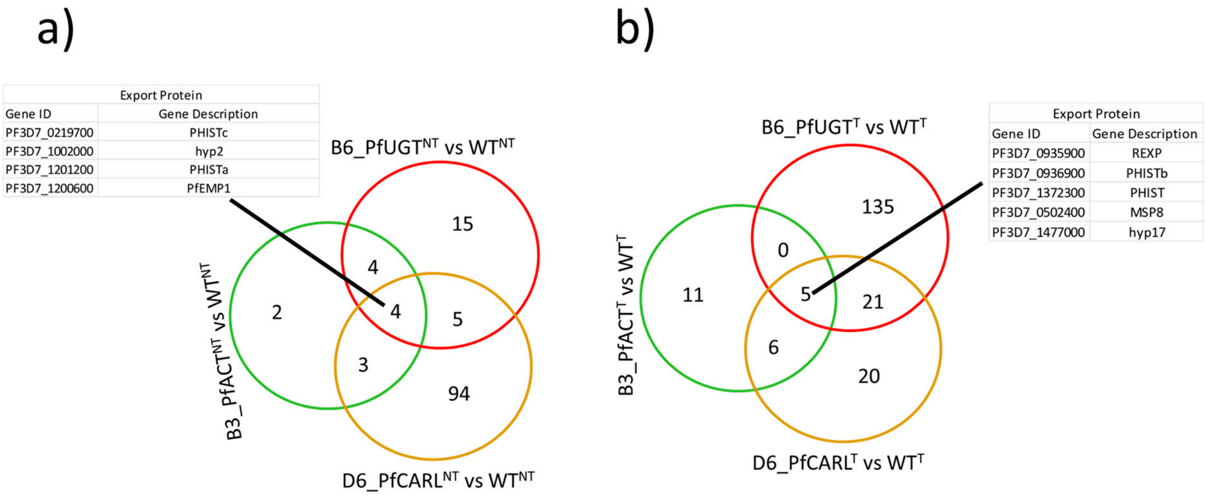

Figure S4. Export protein affected on common mutant parasites in treated and untreated conditions. Veen diagram showing mutation impact on parasites commonly affected gene associated with protein trafficking in:

a) untreated and **b)** GNF179 (100 nM) treated conditions.

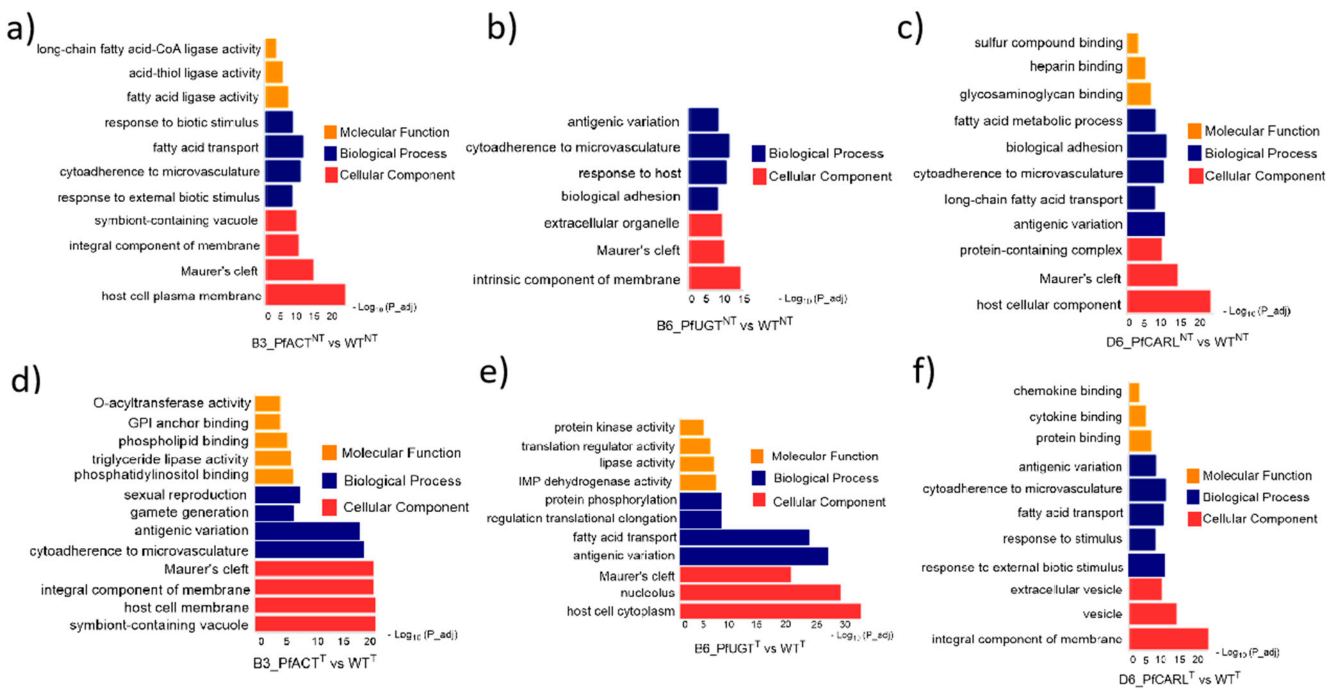

Figure S5. Cellular components, biological process and molecular function disturbed by GNF179 between Wild type and IPZ resistant parasite in GNF179 (100 nM) treated and untreated condition. a) Wild type (WT^{NT}) parasites versus mutant ($B3\_PfACT^{NT}$); **b)** Wild type (WT^{NT}) parasites versus mutant ($B6\_PfUGT^{NT}$), **c)** Wild type (WT^{NT}) parasites versus mutant ($D6\_PfCARL^{NT}$) parasites resistant to IPZ; **d)** Wild type (WT^{T}) parasites versus mutant ($B3\_PfACT^{T}$); **e)** Wild type (WT^{T}) parasites versus mutant ($B6\_PfUGT^{T}$), **f)** Wild type (WT^{T}) parasites versus mutant ($D6\_PfCARL^{T}$) parasites resistant to IPZ.

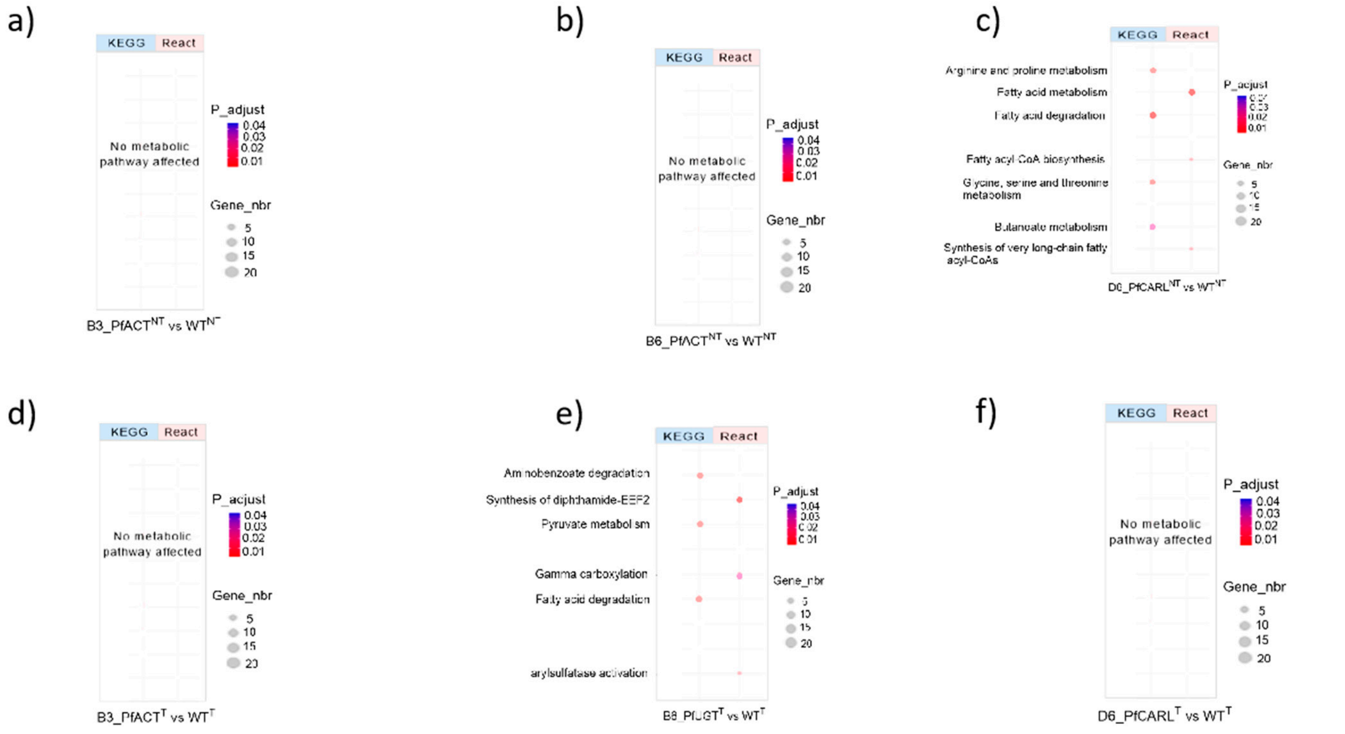

Figure S6. Key metabolic pathways affected by GNF179 between Wild type and IPZ resistant parasite in GNF179 (100 nM) treated and untreated condition. a) Wild type (WT^{NT}) parasites versus mutant ($B3\_PfACT^{NT}$); **b)** Wild type (WT^{NT}) parasites versus mutant ($B6\_PfUGT^{NT}$), **c)** Wild type (WT^{NT}) parasites versus mutant ($D6\_PfCARL^{NT}$) parasites resistant to IPZ; **d)** Wild type (WT^{T}) parasites versus mutant ($B3\_PfACT^{T}$); **e)** Wild type (WT^{T}) parasites versus mutant ($B6\_PfUGT^{T}$), **f)** Wild type (WT^{T}) parasites versus mutant ($D6\_PfCAR^{T}$) parasites resistant to IPZ.
